# Association between diabetes and causes of dementia: Evidence from a clinicopathological study

**DOI:** 10.1590/1980-57642016dn11-040010

**Published:** 2017

**Authors:** Maria Niures Pimentel dos Santos Matioli, Claudia Kimie Suemoto, Roberta Diehl Rodriguez, Daniela Souza Farias, Magnólia Moreira da Silva, Renata Elaine Paraizo Leite, Renata Eloah Lucena Ferretti-Rebustini, Carlos Augusto Pasqualucci, Wilson Jacob, Lea Tenenholz Grinberg, Ricardo Nitrini

**Affiliations:** 1 Department of Neurology, University of São Paulo Medical School , São Paulo, SP, Brazil.; 2 Division of Geriatrics, University of São Paulo Medical School, São Paulo, SP, Brazil.; 3 Aging Brain Study Group from the University of São Paulo Medical School - LIM/22, São Paulo, SP, Brazil.; 4 Medical-surgical Nursing Department, University of São Paulo School of Nursing, São Paulo, SP, Brazil.

**Keywords:** Alzheimer's disease, Diabetes mellitus, vascular dementia, neuropathology, clinicopathologic study, doença de Alzheimer, diabetes mellitus, demência vascular, neuropatologia, estudo clinicopatológico

## Abstract

**Background:**

Diabetes mellitus is a risk factor for dementia, especially for vascular dementia (VaD), but there is no consensus on diabetes as a risk factor for Alzheimer's disease (AD) and other causes of dementia.

**Objective:**

To explore the association between diabetes and the neuropathological etiology of dementia in a large autopsy study.

**Methods:**

Data were collected from the participants of the Brain Bank of the Brazilian Aging Brain Study Group between 2004 and 2015. Diagnosis of diabetes was reported by the deceased's next-of-kin. Clinical dementia was established when CDR ≥ 1 and IQCODE > 3.41. Dementia etiology was determined by neuropathological examination using immunohistochemistry. The association of diabetes with odds of dementia was investigated using multivariate logistic regression.

**Results:**

We included 1,037 subjects and diabetes was present in 279 participants (27%). The prevalence of dementia diagnosis was similar in diabetics (29%) and non-diabetics (27%). We found no association between diabetes and dementia (OR = 1.22; 95%CI = 0.81-1.82; p = 0.34) on the multivariate analysis. AD was the main cause of dementia in both groups, while VaD was the second-most-frequent cause in diabetics. Other mixed dementia was the second-most-common cause of dementia and more frequent among non-diabetics (p = 0.03).

**Conclusion:**

Diabetes was not associated with dementia in this large clinicopathological study.

## RESUMO.

**Introdução:**

Diabetes mellitus é um fator de risco para a demência, especialmente para a demência vascular (DV), mas ainda não há consenso sobre diabetes como fator de risco para a doença de Alzheimer (DA) e outras causas de demência.

**Objetivo:**

Verificar a associação entre diabetes e demência e sua etiologia neuropatológica em um grande estudo de autópsia. Métodos: Os dados foram coletados do Banco de Encéfalos Humanos do Grupo de Estudos em Envelhecimento Cerebral da FMUSP entre 2004 e 2015. O diagnóstico de diabetes foi relatado por pelos parentes do falecido. A demência clínica foi estabelecida quando CDR ≥ 1 e IQCODE > 3,41. A etiologia da demência foi determinada pelo exame neuropatológico com imuno-histoquímica. A associação de diabetes com probabilidades de demência foi investigada usando regressão logística multivariada.

**Resultados:**

Foram incluídos 1.037 sujeitos, diabetes esteve presente em 279 participantes (27%). A frequência de diagnóstico de demência foi semelhante entre diabéticos (29%) e não diabéticos (27%). Não encontramos associação entre diabetes e demência (OR = 1,22; IC 95% = 0,81-1,82; p = 0,34) na análise multivariada. DA foi a principal causa de demência em ambos os grupos, DV foi a segunda causa em diabéticos. A frequência de outra demência mista foi a segunda causa de demência e mais frequente entre os não diabéticos (p = 0,03).

**Conclusão:**

A diabetes não foi associada à demência neste grande estudo clínico-patológico.

## INTRODUCTION

The number of people affected by diabetes mellitus has been growing worldwide.^1^ In 2015, the International Diabetes Federation estimated that 415 million people aged 20-79 years had diabetes worldwide, and nearly 75% lived in low-to-middle-income countries (LMIC).^1^ The number of diabetics will continue to rise with diabetes set to affect 642 million people by 2040, and this will take place especially in regions where economies are transitioning from low-income to middle-income levels.^1^ The World Alzheimer Report 2015 estimated that 46.8 million people worldwide had dementia in 2015, and 58% of these lived in LMIC.^2^ The proportion of people with dementia living in LMIC will reach 63% in 2030 and 68% in 2050.^2^ Dementia and type 2 diabetes are more prevalent with aging.^1^^,^ 2

Diabetes is a known risk factor for dementia and vascular dementia (VaD), but the association between diabetes and Alzheimer's disease (AD) needs to be better clarified.^3^ Several longitudinal studies have shown that diabetes was associated only with VaD, but not with AD.^4^^-^ 9 Meanwhile, some studies have shown that DM is associated with increased risk of dementia and AD,^10^^-^ 17 while others have found a positive association only in the presence of the apolipoprotein E e4 allele (APOE e4).^18^^,^ 19 However, Akomolafe et al.^20^ showed diabetes was a risk factor for AD, especially when three known AD risk factors were absent: elevated plasma homocysteine level, the APOE e4 allele, or age 75 years or older.

There are fewer autopsy studies than epidemiological investigations. Despite the positive evidence linking diabetes and cerebrovascular neuropathology,^21^^-^ 27 most autopsy studies have not supported the same conclusion regarding the association between diabetes and AD neuropathology^22^^,^ 24 ^,^ 26 ^-^ 30 and some authors described a positive association only in individuals carrying the APOE e4 allele.^21^^,^ 23 ^,^ 31 ^,^ 32 Studies from LMIC regarding the association between diabetes and dementia are scarce.^2^ We recently showed that diabetes was not associated with AD-neuropathology in a large sample from Brazil.^30^ Here, we aimed to expand this finding by examining the association between diabetes and causes of dementia in a large autopsy study.

## METHODS

### Participants.

This cross-sectional study included subjects aged 50 years and older from the Brain Bank of the Brazilian Aging Brain Study Group of the University of São Paulo Medical School (BB-BABSG-FMUSP). Exclusion and inclusion criteria for the BB-BABSG-FMUSP were previously described.^33^ Initially, 1,083 subjects were selected from 2004 to 2015, but incomplete interview or neuropathological data were found in 46 deceased. Therefore, the final sample comprised 1,037 deceased. Signed informed consent obtained from the deceased's next-of-kin (NOK) allowed autopsy and brain donation. The local ethics committee approved this study.

### Clinical information.

A skilled team of gerontologist nurses applied the structured interview to the deceased's NOK. The NOK had to have at least weekly contact with the deceased during the six months prior to death. This structured interview provided information about demographics (age, sex, race, and education) and socioeconomic status, previous diagnosis of diseases (hypertension, dyslipidemia, heart failure, coronary artery disease, and stroke), lifestyle (alcoholism, smoking, sedentary lifestyle), medication intake, as well as cognitive and functional status.^33^ A Brazilian scale classifying subjects into five socioeconomic strata^34^ was applied. Stratum A is the highest income and stratum E the lowest, and this scale was grouped into high (stratum A+B), middle (stratum C) and low (stratum D+E) socioeconomic status for statistical analysis. Diabetes was defined based on the NOK report of diabetes diagnosis during life or the use of any antidiabetic medication. Cognitive status was evaluated by the Clinical Dementia Rating scale (CDR)^35^ and the Informant Questionnaire on Cognitive Decline in the Elderly.^36^ The clinical diagnosis of dementia was established in the presence of CDR ≥ 1 and IQCODE score > 3.41.^37^ The cause of dementia was determined following neuropathological criteria.

### Neuropathological assessment.

The brain was collected within 24 hours of death. One hemisphere was coronally sectioned and snap frozen and the other hemisphere was fixed in 4% buffered paraformaldehyde. Samples from the fixed hemisphere were embedded in paraffin and sectioned to produce slides of following regions: hippocampus and parahippocampal gyrus at the level of the lateral geniculate body, amygdala at the level of mammillary bodies (including the ambiens gyrus), basal ganglia at the level of the anterior commissure, thalamus, midbrain, pons, medulla oblongata, and cerebellum, middle frontal gyrus, middle and anterior temporal gyri, angular gyrus, superior frontal gyrus and anterior cingulate gyrus, and visual cortex. Hematoxylin & eosin (H&E) staining was applied to all sampled regions. Selected sections were immunostained with antibodies against phosphorylated tau (PHF-1, dilution 1:2.000; gift of Prof. Peter Davies, NY), b-amyloid (4G8, dilution 1:10.000; Signet Pathology Systems, Dedham, MA), a-synuclein (EQV, 1:10.000, gift of Kenji Ueda, Tokyo, Japan), and transactivation response DNA-binding protein of 43 kDA (TDP-43; 1:500, Proteintech, Chicago, IL).^33^ Internationally accepted neuropathological criteria and guidelines were used for diagnosing and staging the causes of dementia.^33^

AD-type pathology was scored using the Braak and Braak staging system^38^ and the Consortium to Establish a Registry for AD (CERAD)^39^ criteria. Diagnosis of AD dementia was made in cases that had at least Braak stage III and CERAD moderate score.^40^

The presence of diffuse small-vessel disease in the white matter, of hippocampal sclerosis, and of lacunae, microinfarcts, and infarcts were analyzed semi-quantitatively by H&E staining. Cerebral amyloid angiopathy was investigated using anti b-amyloid immunostaining. The diagnosis of VaD was defined in cases with one large chronic infarct (> 1 cm) or three lacunae in strategic areas (thalamus, frontocingular cortex, basal forebrain and caudate, medial temporal area, and angular gyrus).^37^ AD plus VaD was considered as mixed dementia only when the criteria for AD and VaD were concomitantly present.

Lewy body disease was diagnosed based on criteria described by McKeith et al.^41^ To define dementia secondary to Parkinson's disease, a criterion described by Braak et al.^42^ was applied. The diagnosis of Frontotemporal lobar degeneration was established using the criteria of Mackenzie et al.^43^ The diagnosis of argyrophilic grain disease was established according to Saito et al.^44^ Undefined cause was defined when there was no agreement between the clinical diagnosis of dementia and the neuropathological findings. Other mixed dementia was considered in presence of two or more neuropathologies of dementia, except when AD + VaD were present.

### Statistical analysis.

We used the unpaired *t*-test or Mann-Whitney test for quantitative variables, and the Chi-square or Fisher's exact tests for categorical variables, to investigate the association of demographic and clinical variables with dementia stratified by diabetes status. The association between diabetes and dementia was investigated using multivariate ordinal logistic regression with results adjusted for age, sex, education, hypertension, dyslipidemia, smoking, alcohol use and physical inactivity. We also described the frequency of neuropathological diagnosis by diabetes status among participants with dementia, and compared these frequencies using Chi-square or Fisher's exact tests. Stata 13.0 (College Station, TX: StataCorp LP) was employed for statistical analyses. The significance level was set at 0.05 in two-tailed tests.

## RESULTS

We analyzed data from 1,037 subjects, of which 758 (72%) were non-diabetics and 279 (27%) diabetics. Dementia was determined in 80 diabetics (29%) and 205 (27%) non-diabetics, and no difference was found regarding dementia frequency between these groups (p = 0.24). First, the total sample were stratified by diabetes status, and compared for frequency of dementia diagnosis regarding sociodemographic and clinical variables (Table 1). Participants with dementia, with and without diabetes, were mostly female, had lower educational level, and were older compared to non-demented groups. Black and admixed races were more frequent in diabetics with dementia than diabetics without dementia. A higher frequency of stroke and physical inactivity were found in both demented groups. Hypertension and current smoking were more frequent in non-diabetics without dementia compared to non-diabetics with dementia.

**Table 1:** Sociodemographic and clinical data from diabetics and non-diabetics with and without dementia (n=1,037).

Variable	Non-diabetics		*P*	Diabetics		*p*
	Without dementia	With dementia		Without dementia	With dementia	
	(N = 553)	(N = 205)		(N = 199)	(N = 80)	
Female, n (%)*	260 (47.0)	128 (62.4)	**< 0.01**	101 (50.8)	52 (65.0)	**0.03**
Age (years), mean (SD)†	72.6 (11.8)	80.3 (9.7)	**< 0.01**	71.5 (10.9)	78.5 (9.4)	**< 0.01**
Education (years), mean (SD)£	4.4 (3.7)	3.2 (3.4)	**< 0.01**	4.6 (3.8)	3.2 (3.4)	**< 0.01**
Race, n (%)#			0.09			**0.03**
• White	336 (60.8)	120 (58.5)		132 (66.3)	40 (50)	
• Black	104 (18.8)	53 (25.9)		33 (16.6)	20 (25.0)	
• Admixed	99 (17.9)	30 (14.6)		31 (15.6)	20 (25.0)	
• Yellow	14 (2.5)	2 (1.0)		3 (1.5)	0 (0)	
Socioeconomic class, n (%)#			0.41			0.22
• High	162 (29.3)	73 (35.6)		57 (28.6)	26 (32.5)	
• Middle	260 (47.1)	86 (42.0)		106 (53.3)	38 (47.5)	
• Low	130 (23.6)	46 (22.4)		36 (18.1)	16 (20.0)	
Hypertension, n (%)*	354 (64.0)	102 (49.8)	**< 0.01**	165 (82.9)	59 (73.8)	0.08
Dyslipidemia, n (%)*	37 (6.7)	6 (2.9)	0.05	41 (20.6)	9 (11.3)	0.06
Coronary artery disease, n (%)*	118 (21.3)	33 (16.1)	0.11	59 (29.6)	15 (18.8)	0.06
Heart failure, n (%)*	94 (17.0)	27 (13.2)	0.20	43 (21.6)	12 (15.0)	0.21
Stroke, n (%)*	57 (10.3)	52 (25.4)	**< 0.01**	34 (17.1)	24 (30.0)	**0.02**
Physical inactivity, n (%)*	243 (49.4)	136 (82.4)	**< 0.01**	107 (57.8)	53 (88.3)	**< 0.01**
Current smoking, n (%)*	169 (30.6)	39 (19.0)	**< 0.01**	40 (20.1)	18 (22.5)	0.78
Current alcohol use, n (%)*	139 (25.1)	39 (19.0)	0.10	41 (20.6)	14 (17.5)	0.67

* Chi-square test;

# Fisher's exact test

† Student's t-test;

£ Mann-Whitney Test.

Second, only diabetics and non-diabetics with dementia data were compared. Similar frequencies were found between them regarding age (p = 0.12), sex (p = 0.79), education (p = 0.88), race (p = 0.17), socioeconomic status (p = 0.70), coronary artery disease (p = 0.72), stroke (p = 0.52), heart failure (p = 0.83), physical inactivity (p = 0.90), current smoking (p = 0.60), and alcohol use (p = 0.90). Hypertension (p < 0.01) and dyslipidemia (p < 0.01) were more frequent in demented diabetics than in non-diabetics with dementia. There was a higher frequency of individuals with severe dementia (CDR = 3) among non-diabetics (p = 0.03), and with mild (CDR = 1) and moderate dementia (CDR = 2) among diabetics. Non-diabetics with dementia died with highest mean IQCODE scores (4.43; SD:0.48; p = 0.04) (Table 2).

**Table 2:** CDR and IQCODE in diabetics and non-diabetics with dementia (n = 285).

Variables	Non-diabetics with dementia n = 205	Diabetics with dementia n = 80	*p*
CDR, n (%)#			0.03
• 1	45 (22.0)	26 (32.5)	
• 2	42 (20.5)	22 (27.5)	
• 3	118 (57.5)	32 (40.0)	
IQCODE, mean (SD)*	4.43 (0.48)	4.30 (0.45)	0.04

SD: standard deviation; CDR: Clinical Dementia Rating; IQCODE: Informant Questionnaire on Cognitive Decline in the Elderly;

* Mann-Whitney test;

# Chi-square test.

Diabetes was not associated with dementia (OR:1.22; 95%CI:0.81-1.82; p = 0.34) on multivariate ordinal logistic regression adjusted for age, sex, race, education, hypertension, dyslipidemia, smoking, physical inactivity and alcohol use (Table 3).

**Table 3:** Association between clinical dementia and diabetes mellitus status (n = 1,037).

DM	Clinical dementia n (%)		Simple regression OR (95% CI)	Multivariate regression* OR (95% CI)
	No	Yes		
No	553 (73.0)	205 (27.0)	1.00 (reference)	1.00 (reference)
Yes	199 (71.3)	80 (28.7)	1.08 (0.80-1.47)	1.22 (0.81-1.82)
*p*			0.602	0.340

OR: odds ratio; CI: confidence interval.

* Multivariate ordinal logistic regression adjusted for age, sex, race, education, hypertension, dyslipidemia, physical inactivity, smoking, and alcohol use.

Among participants with dementia, AD was the main neuropathological cause of dementia in both groups (Table 4). Only other mixed dementia was more frequent in non-diabetics with dementia (p = 0.03). The distribution of causes of dementia differed between diabetics and non-diabetics. The second-most-common cause of dementia in diabetics was VaD, but in non-diabetics was other mixed dementia. Undefined cause of dementia was similar between diabetics and non-diabetics (Table 4).

**Table 4:** Neuropathological causes of dementia in diabetics and non-diabetics (n = 285).

Neuropathology, n %	Non-diabetics with dementia, n (%) N = 205	Diabetics with dementia, n (%) N = 80	*P*
AD**	52 (25.4)	24 (30.0)	0.52
VaD**	22 (10.7)	14 (17.5)	0.65
Mixed dementia (AD +VaD)*	15 (7.3)	03 (3.8)	0.30
Other Mixed NP**	46 (22.4)	09 (11.3)	0.03
Other single NP**	27 (13.2)	11 (13.8)	0.95
Undefined**	43 (21.0)	19 (23.8)	0.73

AD: Alzheimer's disease; NP: neuropathology; VaD: vascular dementia.

** Chi-square test;

* Fisher's exact test.

## DISCUSSION

This study did not find an association between diabetes and dementia in a large sample of subjects submitted to autopsy. All diabetics and non-diabetics with dementia were older, predominantly women, had lower education, and a higher frequency of stroke and physical inactivity. Older age, low education, female sex, physical inactivity and stroke have been considered risk factors for dementia.^2^^,^ 45 ^-^ 48 Participants with diabetes died at mild and moderate stages of dementia. They had more comorbidities, exhibiting more hypertension and dyslipidemia; therefore, they might have died before dementia had reached more advanced clinical stages.

The prevalence of dementia among non-diabetics (27%) and diabetics (29%) was lower in our study compared with other reports.^22^^,^ 26 ^,^ 27 Arvanitakis et al.^22^ found dementia in 44.2% of non-diabetics and in 50% of diabetics (p = 0.52). Ahtiluoto et al.^26^ reported a very high frequency of dementia, present in 65.9% of non-diabetics and 60.0% of diabetics (p = 0.381) with mean age of 88 years. Abner et al.^27^ reported dementia in 38.2% of non-diabetics and 39.1% of diabetics (p = 0.69), also with a mean sample age of 88.7 years. However, the study by Alafuzoff et al.^23^ found a higher frequency of dementia in the non-diabetic group (p < 0.01) of 31% in non-diabetics and 21% in diabetics, but with a mean age similar to the present study. Peila et al.^21^ described an increased risk for the development of dementia in diabetics, whereas the study by Ahtiluoto et al.^26^ reported double the incidence of dementia in this group.

AD was the main cause of dementia in both groups, showing the importance of this neurodegenerative disease in older people even in the presence of vascular risk factors. VaD was the second-most-common cause only in diabetics. Other mixed neuropathology of dementia was more frequent and the second-most-frequent cause of dementia in non-diabetics. The difference in cause of dementia distribution can probably be explained by diabetics being a group with more vascular risk factors, showing the important role of diabetes in cerebrovascular disease. DM was not associated with increased frequency of any particular type of dementia in the present study, unlike cohort studies that have shown an increased risk for AD,^10^^-^ 17 ^,^ 19 ^,^ 49 VaD,^5^^-^ 9 ^,^ 18 AD and VaD,^11^^,^ 21 ^,^ 26 ^,^ 50 or for mixed AD and VaD^11^^,^ 21 in diabetic patients Diabetes increases the risk for cerebral small-vessel disease, cerebral infarcts, vascular cognitive decline and VaD.^3^^,^ 51 ^,^ 52 The role of diabetes with regard to AD remains controversial, where cerebrovascular disease is likely the main mechanism involved and diabetes possibly decreases brain resilience, but does not directly cause AD.^3^

The present study has several strengths: a) the large sample size from a LMIC with low educational level among subjects, in contrast with other studies; b) autopsy studies allow for the direct measurement of neuropathological lesions, untangling the pure association between DM and dementia neuropathology; c) neuropathologists were blinded to clinical information. However, our study has some limitations: a) this is a cross-sectional study and therefore limited in determining causality of diabetes for dementia; b) all clinical data, including diabetes diagnoses, were obtained in NOK; c) the clinical diagnosis of dementia was established only after death based on NOK information. However, the accuracy of our clinical interview was tested previously and showed good reliability.^53^

In conclusion, we did not find an association between diabetes and dementia frequency or any neuropathological cause in a large sample with low educational level in a LMIC. Further studies are necessary to better clarify these questions, especially longitudinal autopsy studies in LMIC.
